# Does DHE imaging shed insight into the inaccuracy of EKGs?

**DOI:** 10.1186/1532-429X-11-S1-P190

**Published:** 2009-01-28

**Authors:** Wadih Nadour, Robert W Biederman

**Affiliations:** Allegheny General Hospital, The Gerald McGinnis Cardiovascular Institute, Pittsburgh, PA USA

**Keywords:** Ischemia, Cardiomyopathy, Cardiovascular Magnetic Resonance, False Positive Result, Late Gadolinium Enhancement

## Background

The presence of Q wave on EKG remains in wide clinical use to identify people with prior myocardial infarct (MI), in particular transmural infarct. We hypothesized that standard assessment of infarct presence relying on EKG is both insensitive and inaccurate, especially as compared to CMR DHE assessment, regardless of the DHE pattern (ischemic versus non-ischemic).

## Objectives

The purpose of the study is to relate EKG defined scar (Q waves) as compared to late gadolinium enhancement (DHE) cardiovascular magnetic resonance (CMR) defined scar independent of the underlying pathology.

## Methods

We reviewed a total of 106 unselected, consecutive patients who underwent CMR with DHE and had an EKG performed within 1 day of CMR between 2006 and 2008 at our institution. Non-ischemic, infiltrative and other cardiomyopathic patients were not excluded. EKGs performed on the same day or the day prior to the test were screened for the presence of a Q wave and compared to those with a positive DHE regardless of the myocardial post-gadolinium pattern. Then we divided the patients into three subgroups: 1) one group with only positive DHE (DHE**+**) pattern of ischemia 2) the other group with other DHE (DHE**+/-**) patterns or 3) negative DHE (DHE-). Furthermore, we divided the ischemic group (DHE**+**) into transmural vs. endocardial post-gadolinium patterns.

## Results

While 58/106 (45%) patients were either DHE**+** orDHE**+/-,** Q waves were present in only 24/106 patients (22%). However, of the DHE**+** or DHE**+/-** patients, only 22 (20%) had an ischemic DHE pattern, representing little overlap between presence of DHE**+** and finding of Q waves on EKG. The sensitivity and specificity of EKG to detect infarct were 63% and 90%, respectively. The positive predictive value was 73% and negative predictive value was 85%. In the subgroup of patients divided into transmural infarcts (15) and endocardial infarcts (7), the sensitivity was 60% and 57%, respectively. However, of 10/24 (41%) patients with Q waves on EKG, 50% were either DHE- (5/10) or DHE**+/-** (5/10)**.** The latter group included 3 viral cardiomyopathy, 1 infiltrative disease and 1 hypertrophic cardiomyopathy.

## Conclusion

EKG remains the most widely used method in clinical practice to identify patients with prior infarcts based on the presence of Q waves; however it has a low sensitivity compared to CMR DHE defined scar. This sensitivity does not appreciably change with the presence of transmural versus subendocardial infarcts. Importantly, a significant number of Q waves have no ischemic etiology, indicating a high percentage of false positive results by EKG.

